# Redistributing deaths by ill-defined and unspecified causes on cancer mortality in Brazil

**DOI:** 10.11606/s1518-8787.2021055003319

**Published:** 2021-12-01

**Authors:** Alessandro Bigoni, Amanda Ramos da Cunha, José Leopoldo Ferreira Antunes

**Affiliations:** I Universidade de São Paulo Faculdade de Saúde Pública São Paulo SP Brasil Universidade de São Paulo. Faculdade de Saúde Pública. São Paulo, SP, Brasil; II Harvard T.H. Chan School of Public Health Boston MA United States of America Harvard T.H. Chan School of Public Health. Fulbright Fellow. Boston, MA, United States of America; III Universidade Federal do Rio Grande do Sul Faculdade de Odontologia Porto Alegre RS Brasil Universidade Federal do Rio Grande do Sul. Faculdade de Odontologia. Porto Alegre, RS, Brasil

**Keywords:** Chemical Waste, Hazardous Waste, Product Labeling, Substances, Products and Materials Transportation

## Abstract

**OBJECTIVE:**

to discuss the impact four different redistribution strategies have on the quantitative and temporal trends of cancer mortality assessment in Brazil.

**METHODOLOGY:**

This study used anonymized and georeferenced data provided by the Brazilian Ministry of Health (BMoH). Four different approaches were used to conduct the redistribution of ill-defined deaths and garbage codes. Age-standardized mortality rates used the world population as reference. Prais-Winsten autoregression allowed the calculation of region, sex, and cancer type trends.

**RESULTS:**

Death rates increased considerably in all regions after redistribution. Overall, Elisabeth B. França’s and the World Health Organization methods had a milder impact on trends and rate magnitudes when compared to the Global Burden of Disease (GBD) 2010 method. This study also observed that, when the BMoH dealt with the problem of redistributing ill-defined deaths, results were similar to those obtained by the GBD method. The redistribution methods also influenced the assessment of trends; however, differences were less pronounced.

**CONCLUSIONS:**

Since developing a comparative gold standard is impossible, matching global techniques to local realities may be an alternative for methodological selection. In our study, the compatibility of the findings suggests how valid the GBD method is to the Brazilian context. However, caution is needed. Future studies should assess the impact of these methods as applied to the redistribution of deaths to type-specific neoplasms.

## INTRODUCTION

Preventive and therapeutic interventions require constant epidemiological surveillance. Monitoring the magnitude and variation of cancer mortality over time and space allows the evaluation of access to and quality of health services, as well as recognizing risk factors, planning health programs, and setting research priorities^1,2^ Cancer mortality surveillance is even more needed in the absence of accurate and comprehensive incidence data, common in many countries^3^.

Mortality data reliability is a critical aspect of epidemiological studies on cancer. The under-registration of deaths (low coverage of the information system), a high proportion of deaths classified as due to unspecified causes^4^, and the inadequate reporting of immediate or mediate conditions as the underlying cause of death (usually referred to as “garbage codes”)^5^ are the main problems affecting the reliability of mortality data. Both the low coverage and a high proportion of garbage codes affect the Mortality Information System in Brazil^6^.

The quality of mortality data in Brazil increased in recent decades^7^, but significant problems remain^7^. The coverage of the country’s information system is heterogeneous across the states. Economically developed regions, such as the South and Southeast, have coverages comparable to that of richer countries, increasing the system’s overall reliability at the national level. However, in the more impoverished Northeast and North regions, several states reported less than 80% of their deaths to the country’s information system during the 1990s and 2000s. A recent global health study on cancer mortality in high- and middle-income countries excluded Brazilian data due to the insufficient coverage of the mortality information system during this period^3^.

The Global Burden of Disease 2016 study rates Brazil four out of five stars, indicating a 65% to 85% overall percentage of well-certified deaths – a stark contrast to the three stars (35%~65%) the country’s Northern and Northeastern regions received^8^.

Concerns about the information system’s limitations are recurrent in epidemiological studies on mortality, which includes cancer^5,9,10^. Several statistical techniques to correct this problem were reported, resulting in a variety of methods for the same purpose^11^. Primary global information sources, such as the World Health Organization (WHO) and the Global Burden of Disease, applied these methods, and performed different corrections^2,14^. These techniques consisted of redistributing deaths by unspecified causes among the underlying causes of death (or specific types and subtypes of cancer), according to different conceptual frameworks and methodological complexity. However, their differential impact on the results has been little explored. This study aims to discuss the impact on the magnitude and temporal mortality trends of four different redistribution strategies that have been used to assess cancer mortality in Brazil.

## METHODS

### Data

This study gathered data freely provided by the mortality information system in the Brazilian Ministry of Health website. The study period starts in 1996, when the information system adopted the 10^th^ revision of the International Classification of Diseases (ICD-10)^15^. The study period ended in 2017, which is the most recent year with information already available.

Death certificates provide information on the deceased’s sex, age, the underlying cause of death, and place of residence. The Brazilian Institute of Geography and Statistics supplied the number of inhabitants for each town, sex, and age groups. The demographic information relates to the 2000 and 2010 censuses. Estimates were calculated for the remaining years using the exponential growth rate based on two points in time: *R* = *In* (*Pl*/*Pf*)/*Ny*; where *R* is the growth rate for a given municipality, sex and age group, *Pl* and *Pf* arethe last and first measurements in the available period, respectively, and *Ny* is the period range in years. This estimation technique allows for smoother population estimates reducing fluctuations in mortality trends.

To reduce data granularity, we assigned each town to its respective macroregion. Macroregions are the broadest regional divisions in the country: North, Northeast, Southeast, South, and Center-West. We used the built-in *icd10* Stata command to generate a dummy variable which signaled the deaths by cancer. When selecting the ICD-10 codes used as targets, we used the list provided by the Global Burden of Disease (GBD) 2010 study (Box)^14^. The data were subsequently aggregated, resulting in the total number of cancer deaths by macroregion, sex, and age group.

**Box:** Target codes for all cancers according to the ICD-10.

Targets
C00-C139, C15-C259, C30-C349, C37-C388, C40-C419, C43-C459, C47-C549, C56-C578, C58-C580, C60-C638, C64-C679, C680-C688, C69-C758, C81-C866, C88-C969, D001-D002, D010-D013, D020-D023, D03-D069, D070-D072, D074-D075, D090, D092-D093, D098, D100-D107, D11-D129, D130-D137, D140-D143, D15-D169, D22-D249, D260-D279, D280-D281, D287, D290-D298, D300-D308, D31-D36, D361-D367, D371-D375, D380-D385, D391-D392, D398, D400-D408, D410-D418, D42-D439, D440-D448, D45-D479, D480-D486, D492-D494, D496, K620-K621, K635, N60-N609, N840-N841, N87-N879.

### Redistribution Methods

The GBD 2010 Study used the method proposed in Naghavi et al 2010 to identify garbage codes in death certificates^5^. Garbage codes are codes not considered useful to the analysis of underlying causes of death and should, thus, be redistributed to enhance the validity of the analysis. The Global Burden of Disease 2017 Study expanded the number of ICD-10 codes identified as garbage codes^16^. Each garbage code was assigned to its respective group, as proposed in the GBD 2010. The probability of a certain cause of death to be misclassified as a certain garbage code varies depending on the cause of death. The GBD 2010 estimated the proportion of each death by a garbage code that should be assigned to each specific cause of death by applying a method proposed by Ahern et al 2011^17^. The following formula allows calculating the total amount of deaths attributable to a specific underlying cause:


$$NDc+\sum NDg_{i}*C_{i}$$

Where *NDc* is the number of deaths whose cause was certified as being cancer, *i* is the garbage code group, *NDg*_*i*_ is the total number of deaths by a garbage-code group, and *C* is the coefficient proportion taken from^14^.

In 2014, França et al. (EF method) proposed a different method of redistribution, which focused solely on the 18^th^ chapter of ICD-10^9^. The study investigated the misclassified causes of death and proposed coefficients to guide their redistribution. They built on the WHO’s recommendations (WHO method) for death redistribution, found in the 18^th^chapter which describes the proportion of deaths in the remaining chapters of the ICD-10^18^, according to the following formula:


$$NDr*\left(\frac{NDc}{AD-NDr-NE}\right)$$

Where *NDc* is the certified number of deaths by cancer, *NDr* is the number of deaths found in ICD-10’s 18^th^ chapter (R00-R99), *AD* is the total number of deaths and *NE* is the total number of deaths by external causes. The EF method followed the assumption (verified in a small sample of deaths that underwent verbal autopsy) that cancer deaths have a lower chance of being misclassified than deaths by other diseases, and redistributed only half of the R00-R99 neoplasm deaths following the proportion proposed in the WHO method.

The Brazilian Ministry of Health also developed a method (BMoH method) to correct mortality data^6^. They calculated the correction coefficients by taking into account data from verbal autopsies and medical record reviews performed in the country. Although Ministry of Health has not fully disclosed their data, they provided the total values of redistributed deaths by neoplasms by macro-region, age group, and sex for the years 2000 to 2013. Within these four scenarios, we also proportionally redistributed deaths of unknown sex and age according to region and year of death.

### Analysis

We calculated age-standardized mortality rates (ASMR) for the four scenarios, in each year, macro-region, and sex. The standardization of rates used the reference population defined by the WHO^19^. The assessment of trends used the Prais-Winsten method for generalized linear regression, with log-transformed ASMRs as the outcome variable, and year of death as the covariate. This method takes into consideration the first-order serial autocorrelation that affects timely ordered measurements of social processes. The resulted β1 and its confidence intervals (β1_*lower*_ and β1_*upper*_ ) were used to calculate the annual percentage change (APC) using the formula described by Antunes and Waldman: *APC*% = (-1 + 10^1^) * 100. Similarly, the confidence intervals of the APC can be calculated by substituting β by β1_*lower*_ and β1_*upper*_ in the same formula^20^. The trend is increasing if the resulting APC and its confidence interval are positive; the trend is decreasing when they are negative; the trend is stationary when the confidence interval includes the zero.

The assessment of the impact of redistributing deaths used three measurements. The first two were the initial and final magnitude of mortality, where the initial magnitude is the average death rates for the first three years of the study period and the final magnitude is the average death rates for the last three years. The third measurement was the APC, as defined above, which refers to the trend of mortality for each scenario. Both the death rates and the APC were compared in terms of the rate ratios, considering figures related to certified death rates as the reference and figures related to each redistribution method as the comparison category.

The steps detailed above are reproducible through the user-written Stata command *charon,* which can be downloaded to Stata using the command *ssc install charon*. We provided the dataset used for this study in Additional file 2. All the steps of this analysis used Stata 15.1 (College Station, Texas, 2019).

## RESULTS

Cancer mortality was on the increase in the more impoverished North and Northeast Brazilian regions, whereas rates declined in the wealthier South and Southeast regions (Figure). Table 1 depicts cancer mortality across the regions in the initial and final years of the monitoring, as assessed by the different methods of redistribution. It also depicts differences in trends over the study period.

**Figure:**
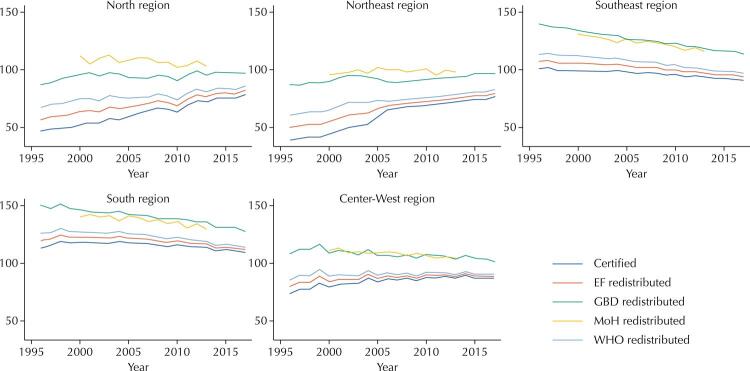
Time series of cancer mortality in Brazil, 1996–2017, by macroregions. Certified, EF redistributed, and GBD redistributed death rates as adjusted for age and gender.

**Table 1:** Certified and redistributed cancer mortality in Brazil, 1996–2017. Age and genderadjusted rates (per 100,000 inhabitants) and Annual Percentage Change by macroregions.

	North	Northeast	Southeast	South	Center-West
Initial magnitude					
Certified	48.32	40.80	100.91	116.40	76.93
EF redistributed	58.94	51.63	107.07	122.15	82.82
WHO redistributed	69.55	62.47	113.23	127.91	88.71
GBD redistributed	89.56	87.83	137.81	149.87	111.23
BMoH redistributed^a^	108.94	96.88	129.42	141.18	116.50
Final magnitude					
Certified	76.77	75.35	92.13	110.99	87.79
EF redistributed	80.54	78.47	95.14	113.27	89.31
WHO redistributed	84.31	81.59	98.15	115.55	90.82
GBD redistributed	97.20	96.77	115.37	130.12	103.64
BMoH redistributed^a^	104.85	97.75	117.12	131.72	105.43
APC					
Certified	2.51 [2.30; 2.72] ↑	3.27 [2.30; 4.25] ↑	-0.48 [-0.54; -0.41] ↓	-0.24 [-0.44; -0.03] ↓	0.68 [0.46; 0.90] ↑
EF redistributed	1.62 [1.46; 1.78] ↑	2.23 [1.70; 2.76] ↑	-0.64 [-0.71; -0.57] ↓	-0.38 [-0.57; -0.19] ↓	0.37 [0.23; 0.50] ↑
WHO redistributed	0.92 [0.72; 1.12] ↑	1.40 [1.09; 1.71] ↑	-0.78 [-0.87; -0.69] ↓	-0.52 [-0.70; -0.34] ↓	0.09 [-0.01; 0.19] ↔
GBD redistributed	0.32 [0.05; 0.60] ↑	0.43 [0.10; 0.76] ↑	-0.93 [-0.96; -0.90] ↓	-0.71 [-0.81; -0.62] ↓	-0.40 [-0.51; -0.29] ↓
BMoH redistributed^a^	-0.43 [-0.75; -0.11] ↓	0.09 [-0.17; 0.35] ↔	-0.84 [-0.98; -0.69] ↓	-0.60 [-0.72; -0.48] ↓	-0.49 [-0.60; -0.38] ↓

^a^ It refers to the period from 2000 to 2013.

↑ Increasing trend; ↓ Decreasing trend; ↔ Stationary trend; EF: redistribution method proposed by França et al. (2014)^9^; GBD: redistribution method proposed by Lozano et al.^14^ (GBD 2010); APC: annual percentage change.

The South region had the highest rates in all scenarios, and on all periods, while the Northeast had the lowest. In the Center-West region, certified death rates were on the increase; the same was observed after redistribution of deaths by the EF method. However, the trend was stationary after redistribution by the WHO method, and it was decreasing when redistribution of deaths used the methods proposed by the GBD study and the Brazilian Ministry of Health (Table 1). Overall, redistributing the deaths by ill-defined causes resulted in lower figures of APC than the mortality exclusively referred to cancer deaths with certified underlying causes, which suggests that the mortality information system may have improved over the study period.

In the first years of the trend, the impact of the EF redistribution was higher in the North region, where the rates suffered a 22% increase compared to certified death rates, and lower in the South, where the increase was only of 5%. Expectedly, the WHO redistributed scenario yielded a rate two times higher than the EF scenario. The GBD redistribution method had a higher impact in the Northeast and a lower impact in the South, corresponding to an increase of, respectively, 115% and 29% in the rates (Table 2).

**Table 2:** Cancer mortality in Brazil, 1996–2017, by macroregions. Impact of redistributing deaths by unspecified and ill-defined causes.

	North	Northeast	Southeast	South	Center-West
RR of initial magnitude					
Certified	1.00	1.00	1.00	1.00	1.00
EF redistributed	1.22	1.27	1.06	1.05	1.08
WHO redistributed	1.44	1.53	1.12	1.10	1.15
GBD redistributed	1.85	2.15	1.37	1.29	1.45
BMoH redistributed^a^	2.03	2.04	1.30	1.19	1.37
RR of final magnitude					
Certified	1.00	1.00	1.00	1.00	1.00
EF redistributed	1.05	1.04	1.03	1.02	1.02
WHO redistributed	1.10	1.08	1.07	1.04	1.03
GBD redistributed	1.27	1.28	1.25	1.17	1.18
BMoH redistributed^a^	1.72	1.61	1.27	1.18	1.27
APC of RR of all years					
EF redistributed	-0.77 [-0.97; -0.57] ↓	-1.02 [-1.50; -0.54] ↓	-0.14 [-0.18; -0.09] ↓	-0.14 [-0.16; -0.12] ↓	-0.29 [-0.41; -0.17] ↓
WHO redistributed	-1.38 [-1.72; -1.03] ↓	-1.80 [-2.64; -0.95] ↓	-0.26 [-0.35; -0.17] ↓	-0.27 [-0.32; -0.23] ↓	-0.55 [-0.78; -0.32] ↓
GBD redistributed	-2.07 [-2.45; -1.68] ↓	-2.73 [-3.83; -1.61] ↓	-0.46 [-0.54; -0.39] ↓	-0.49 [-0.64; -0.35] ↓	-1.07 [-1.37; -0.77] ↓
BMoH redistributed^a^	-2.89 [-3.17; -2.61] ↓	-3.53 [-4.60; -2.44] ↓	-0.39 [-0.54; -0.23] ↓	-0.28 [-0.39; -0.17] ↓	-1.13 [-1.40; -0.85] ↓

^a^ It refers to the period from 2000 to 2013.

EF: distribution method proposed by França et al.^9^; GBD: distribution method proposed by Lozano et al.^14^ (GBD 2010); APC: average percentage change.

The impact of the distributions was lower during the last years of the trend. The EF redistribution method added no more than 4% in all regions, while the GBD redistribution method had its highest impact in the Northeast (a 28% increase in rates) and its lowest impact in the South (a 17% increase in rates). Table 2 depicts the annual percentage change of rate ratios comparing redistributed and certified death rates. The rate ratios for all redistribution methods consistently decreased in the five regions, which also suggests that deaths by unspecified causes and garbage codes shrunk and fewer cases had to be redistributed over the years.

## DISCUSSION

This study assessed the impact of different methods of redistributing deaths by ill-defined causes on cancer mortality. Death rates increased considerably in all regions after performing the redistribution. Overall, the EF and WHO methods had a milder impact on trends and magnitudes of rates when compared to the method used in the GBD study. This study also observed that when the Brazilian Ministry of Health dealt with the problem of redistributing ill-defined deaths, the results were similar to those obtained by the GBD method. The redistribution methods also influenced the assessment of trends; however, differences in the annual percent change were less pronounced. These are the main results reported here.

To our knowledge, this is the first study systematically assessing the impact of redistribution methods on cancer mortality in Brazil. Nevertheless, a previous study on cancer mortality in Brazilian state capitals made a brief discussion about the importance of correcting the estimates by the WHO method and offered a graphic display of the differences obtained at the country-level^11^. In addition to considering the whole country (not only the state capitals), this study included several methods of redistribution, assessed differences in magnitude and trends across all regions, and concluded that the direct appraisal of certified mortality could lead to a severe underestimation of cancer mortality.

Differences in the impact of the methods reflect specific features of the methods that attempt to deal with the low-quality of death notification. The GBD study relied on constant (i.e., not dependent on age) coefficients to redistribute deaths by ill-defined causes. The EF and WHO methods, on the other hand, used the proportion of deaths with certified causes (which varies across the different age groups) to redistribute ill-defined deaths.

The methods assessed here also differ in the selection of cases to redistribution. The EF and WHO methods exclusively redistribute deaths whose certified underlying cause was ill-defined or unknown, explicitly referring to the 18^th^ chapter of ICD-10. The GBD study expanded the selection criteria by applying the concept of “garbage codes”^5^ to other ICD-10 chapters, mainly referring to immediate and intermediate causes of death, which should not have been selected as the underlying cause. The BMoH method gave priority to correcting the sub-notification of deaths. These methodologic characteristics may have contributed to the differential impact of the redistribution, as compared with the certified mortality, and the higher increment in magnitude resulting from the GBD and BMoH methods.

The impact of the methods also varies according to age. Older age groups have a higher proportion of deaths by ill-defined causes^7,21^. Therefore, they also have a higher input of redistributed deaths and are susceptible to the inaccuracy that may be inherent in the methods. Thus, it is possible that using constant coefficients to redistribute deaths by garbage codes overestimates mortality for the age groups with a high proportion of adequate cause-of-death certification, and, vice-versa, underestimates it for the older age groups. This argument stands for the need to be careful when estimating cancer mortality for specific age groups.

The visual inspection of the series suggests that the impact of all redistribution methods on cancer mortality has decreased over time. This observation may be due to an improvement in the quality of mortality data, characterized by a reduction in the deaths certified as due to ill-defined causes and garbage codes. This finding is consistent with the conclusions in Mikkelsen et al. 2015, who applied a composite index to assess vital statistics in Brazil from 1980 to 2002. They concluded that the country’s information systems had consistently progressed^22^. Advances in the quality of civil registration in Brazil had already been reported by Szawarcwald, who highlighted, however, the persistence of significant regional discrepancies in data quality^23^.

The GBD study is an ongoing example of continuous effort in improving the assessment of mortality. Its proponents have modified their method of redistribution since its first publication in 1996^24^. The publication of the method used in the GBD study, including the coefficients for redistributing deaths by ill-defined causes, helped to disseminate its application, due to its reproducibility (GBD 2010)^14^. Subsequently, however, the GBD study updated its method to a more sophisticated modeling, which required complex algorithms, and information not made available by their proponents (GBD 2017)^1^. This difficulty made us apply, in this assessment, the method published by the GBD 2010, which is the main limitation of this study.

The statistical monitoring of cancer mortality is unquestionably relevant to assess population health status and plan health programs and interventions. The quality of the information system has motivated several efforts in public health. These efforts range from the development of advanced methodologies for evaluating the performance of information systems^22,25,26^, to international investments for the direct qualification of registrants^27^. However, the global reality remains far from the ideal, especially in poorer countries^21^. Setel et al 2007. interpreted the persistent failure over the last decades in establishing and maintaining civil registration systems and ensuring that the causes of death are known with precision worldwide as being a “scandal of invisibility”^30^. Thus, the development and research on indirect methods for correcting of correction methodscause-of-death statistics remain crucial.

Given the impossibility of developing a gold standard method for comparison (which would involve investigating the underlying cause of death for each ill-defined case in a given region), the matching of global techniques with those that consider the local reality may be an alternative for the methodology selection. In the present study, the compatibility of the findings obtained by the GBD and BMoH methods suggests the validity of the first concerning the Brazilian context. However, caution is needed in this interpretation, because this study only redistributed ill-defined deaths for all cancers. Future studies should assess the impact of these methods as applied to the redistribution of deaths to type-specific neoplasms.
