# Errata

**DOI:** 10.11606/s1518-8787.2021055003319err

**Published:** 2022-01-11

In the article “**Redistributing deaths by ill-defined and unspecified causes on cancer mortality in Brazil**”, DOI https://doi.org/10.11606/s1518-8787.2021055003319, published on the Revista de Saúde Pública. 2021;55:106, on page 1, where it reads:

**DESCRIPTORS:** Chemical Waste. Hazardous Waste. Product Labeling. Substances, Products and Materials Transportation.

It should read as follows:

**DESCRIPTORS:** Neoplasms, mortality. Data Accuracy. Vital Statistics. Cause of Death.

